# Assessment of Bony Pelvis and Vaginally Assisted Deliveries

**DOI:** 10.1155/2013/763782

**Published:** 2013-04-04

**Authors:** Ulla Korhonen, Pekka Taipale, Seppo Heinonen

**Affiliations:** ^1^Department of Obstetrics and Gynecology, Kuopio University Hospital, P.O. Box 100, 70029 KYS, Finland; ^2^Department of Obstetrics and Gynecology, North-Carelian Central Hospital, Tikkamäentie 16, 80210 Joensuu, Finland; ^3^Department of Obstetrics and Gynecology, Iisalmi Hospital, Riistakatu 21, 74100 Iisalmi, Finland

## Abstract

*Objective*. To evaluate whether pelvic measurements have any association with operative vaginal deliveries and the duration of the second stage of the delivery. *Study design*. A retrospective study of pregnant women at an increased risk of fetal-pelvic disproportion during 2000–2008 in North-Carelian Central Hospital. The mode of the vaginal delivery was chosen to represent the reference standard. The target condition was spontaneous vaginal delivery. Patients were divided into subgroups according to the size of the fetus and also by the parity to evaluate the variability reflecting differences in patient groups. Receiver operating characteristic (ROC) curves were established. *Results*. A total of 226 participants with fetal cephalic presentation delivered vaginally; of these, 184 women delivered spontaneously, and 42 women required operative vaginal delivery with vacuum extraction. There were no clinically or statistically significant differences between the size of the maternal pelvic outlet and the different modes of delivery types within these subgroups. With respect to the pelvic inlet and outlet, the areas under the curve in ROC were 0.566 with the *P* value of 0.18 and 95% confidence interval (CI) of 0.465–0.667 and 0.573 (95% CI: 0.484–0.622; *P* = 0.14). *Conclusions*. The maternal bony pelvic dimensions exhibited virtually no correlation with the need for operative vaginal deliveries.

## 1. Introduction

Cephalopelvic disproportion (CPD) in labour occurs when there is a mismatch between the size of the fetus and the dimensions of the maternal pelvis. The factors which mainly influence the outcome of the delivery can be summarised as the three “Ps” of the labour: passageway, passenger, and power of the uterus [1]. The passageway component of this trinity has been investigated by pelvimetry which measures the maternal bony pelvic dimensions [2], with very little emphasis on its shape or pelvic floor muscles. During the last decades, the use of pelvimetry has been discouraged [3], but at present, no replacing methods to evaluate the maternal pelvis have been introduced. The benefits of vaginal deliveries are well known when no risk factors are present [4] even after previous Cesarean section (CS) [5, 6]. On the other hand, unplanned interventions during labour such as acute or emergency Cesarean sections as well as operative vaginal delivery increase both maternal and fetal morbidities [7] as does a prolonged second stage of the delivery [8]. The safety and the accuracy of the measurements obtained in pelvimetry have improved in the era of the MRI technology [9, 10]. It is also in the interest of the mother and her physician to minimize the number of unplanned interventions during labour.

The purpose of this observational cohort study was to evaluate whether pelvic measurements, especially pelvic outlet, displayed any association with operative vaginal deliveries and the duration of the second stage of the delivery.

## 2. Materials and Methods

This retrospective study was approved by the Ethical Committee of North-Carelian Central Hospital. It investigated Caucasian women, that had been examined by X-ray or MRI pelvimetry during 2000–2008 in North-Carelian Central Hospital. The patients were sent to the hospital antenatal unit from their general health care. Eligibility criteria included that pelvimetric and fetal measurements had been recorded. In the operative delivery group, the criteria were as follows. There were no signs of fetal distress in cardiotocography, inertia was not diagnosed, and there was no malpresentation. Originally, 915 women were screened for possible inclusion, but 429 women were excluded because of breech presentation. A total of 486 patients with the fetus in the cephalic presentation were screened in the study, but those 234 women that went through elective or acute Cesarean section were excluded from the analysis. The clinical indication for pelvimetry was breech presentation, or if the fetus was in cephalic presentation, the indication was suspected cephalopelvic disproportion in clinical examination. The findings that referred to CPD in clinical examination were clinically small pelvis, unengaged presentation, or suspected macrosomia. Pelvimetric measurements were found in all patients, as required by the inclusion criteria. There were 252 participants with fetal cephalic presentation delivered vaginally, of whom 184 women delivered spontaneously and 68 women went through operative vaginal delivery with vacuum extraction. Of this latter group of women, in 26 patients, the vacuum extraction was undertaken primarily because of fetal distress and inertia, and these patients were excluded from the final analysis, leaving 42 women in the operative vaginal delivery group. Thus, the total number of participants evaluated in the final stage of this study was 226.

The obstetric and radiologic data were collected from patients' medical records by the author (UK) and transferred into a commercially available worksheet (Excel, Microsoft 2003, Ireland). The patients were numbered for identification in the order of their pelvimetric examination date. The following pelvimetric parameters were recorded: in the pelvic inlet, anteroposterior (conjugata vera) and transverse diameters and in outlet, interspinous diameter and sagittal diameter from the surface of the pubic symphysis to the surface of the sacrum measured at the spinous level. Pelvic inlet and outlet circumferences were calculated from the pelvic anteroposterior and transverse diameters using the formula (ap + dt × 1.57) [11]. Until the year of 2003, all pelvimetries were performed with an X-ray technique, and from the year 2004 onwards, they were performed with Magnetic Resonance Imaging (MRI). During the transition period, both X-ray and MRI pelvimetries were performed to verify the repeatability of the measurement results [10]. Already at the beginning of 1990, in order to minimize the variability in pelvimetric measurements, they were centralized so that instead of being conducted by several radiologists, they were conducted by trained obstetricians. When the MRI pelvimetry was taken into clinical practice, there was one radiologist with previous experience of MRI pelvimetry, and during a two-year period (2004–2006), three radiologists and further three obstetricians were also trained to measure the images.

In this evaluation of the diagnostic accuracy of the pelvimetry in vaginal deliveries, the mode of the vaginal delivery was chosen to represent the reference standard. The target condition was spontaneous vaginal delivery. Patients were divided into subgroups according to the size of the fetus and also by the parity to evaluate the variability reflecting differences in patient groups.

For statistical analysis, we used SPSS 17.0 (SPSS Inc., 2009, Chicago, IL, USA). Chi-square test was used to investigate the statistical significances. Receiver operating characteristic (ROC) [12] curves were established, and the area under curve (AUC) values with significances were calculated.

## 3. Results

**Figure 1:**
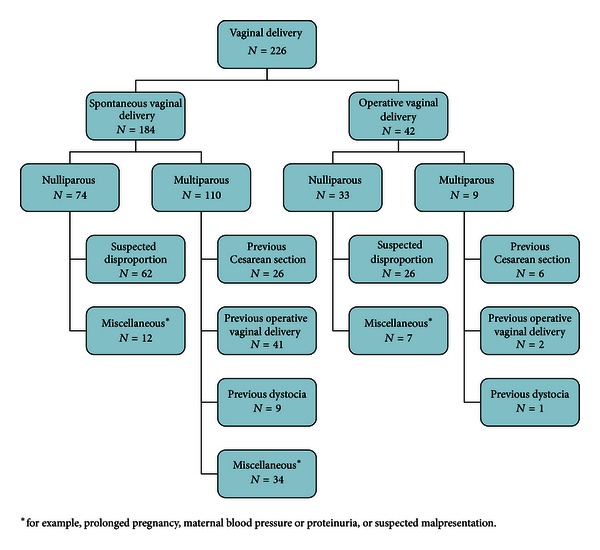
Flow chart of the patients and the reasons why there had been a consultation about the mode of delivery.

Figure 1 shows a flow chart of the 226 patients that were investigated. Pelvimetric measurements were found in all patients, as required by the inclusion criteria. In the spontaneous vaginal delivery group, 40% were nulliparous. Most of the nulliparous patients were sent to maternity clinics consultation because of suspected disproportion. In the multiparous group, 24% had delivered by CS and 37% by operative vaginal delivery. In the operative vaginal delivery group, 79% were nulliparous. Of the nine multiparous patients, six had delivered by CS in their previous pregnancy and two had had previous vaginal operative delivery. The demographic data of these 226 patients subdivided according to the route of delivery are shown in Table 1.

Patients were further subdivided into two subgroups according to the infant's weight and the mode of delivery. The maternal pelvic inlet and outlet sizes and duration of the first and second stages of the delivery by the mode of delivery in infant weight subgroups are shown in Table 2. The mean maternal outlet (±SD) was 3613 (±20) mm in all, 351 (±17) mm in infant weight <3700 g, and 369.5 (±17.7) mm in infant weight ≥3700 g groups. No clinically or statistically significant differences in the pelvic sizes were found between the modes of delivery within the subgroups. Between the subgroups, the size of the maternal pelvic size was 4%-5% larger in the mothers with infant weight ≥3700 g. The duration of the second stage of the delivery was 54 minutes longer (*P* < 0.01) in the operative vaginal delivery group amounting to a 45-minute longer duration (*P* = 0.01) in infant weight <3700 g and 62 minutes longer (*P* < 0.01) in infant weight ≥3700 g group. The one-minute Apgar scores were above 8 in all groups with the exception of those with infant weight less than 3700 g in the operative vaginal group, where the mean of Apgar score at one minute was 7.8 ± 1.8.

**Figure 2:**
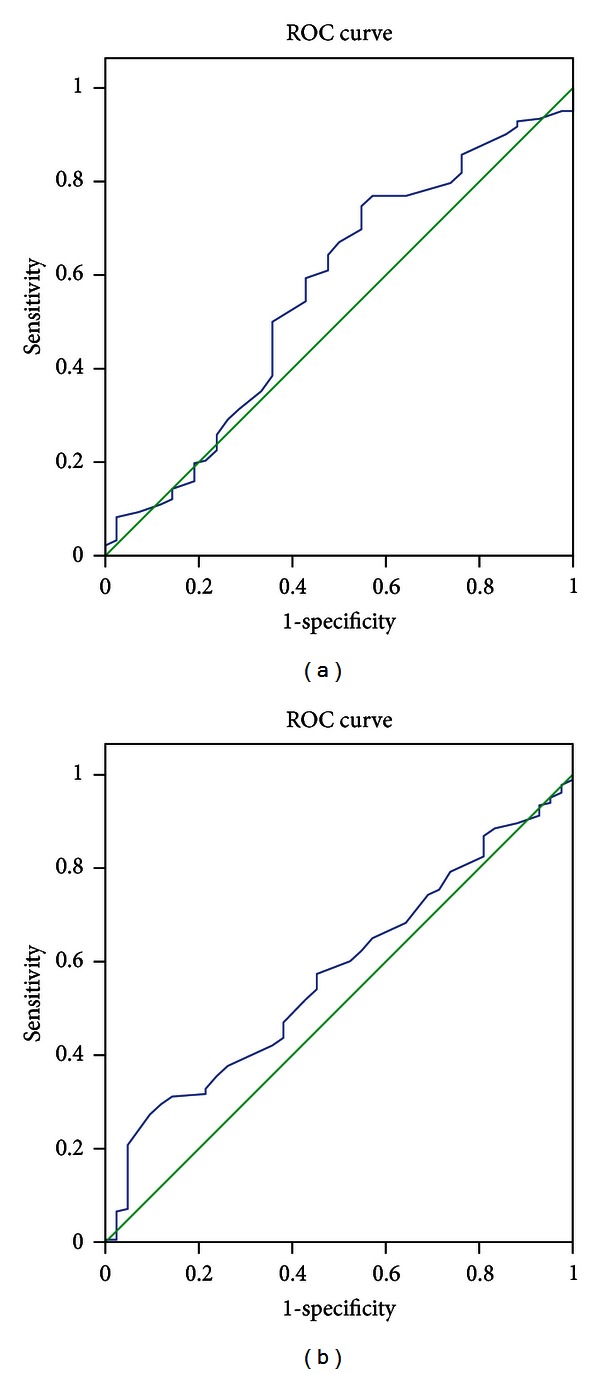
ROC curves of the maternal pelvic inlet and outlet and the mode of the vaginal delivery. Spontaneous vaginal delivery was chosen as the reference result. (a) ROC curve for maternal inlet. The area under curve is 0.566 with the *P* value of 0.18 and 95% confidence interval of 0.465–0.667. (b) ROC curve for maternal outlet. The area under curve is 0.573 with the *P* value of 0.14 and 95% confidence interval of 0.484–0.622.

The receiver operating characteristic curve analysis for pelvic inlet and outlet as a diagnostic test for the mode of vaginal delivery is shown in Figures 2(a) and 2(b). The area under the curve (AUC) for the pelvic inlet was 0.566 with the *P* value of 0.18 and 95% confidence interval (CI) of 0.465–0.667. For pelvic outlet, the AUC was 0.573 (*P* value 0.14; 95% CI 0.484–0.622).

## 4. Conclusions

The main finding of this study was that the maternal bony pelvic dimensions displayed virtually no correlation to the need for operative vaginal deliveries. The indications for intervention in vaginal deliveries were chosen on clinical grounds as evidenced by the fact that there was an association between the duration of the second stage of the delivery and the size of the pelvic outlet. If the delivery had reached the second stage, it was probable that the uterine “power” played a more significant role in the overall outcome than either the “passageway” or the “passenger” [1]. On the other hand, pelvic floor muscles, the three-dimensional shape of the bony pelvis, or other soft tissues were not taken into account.

The pelvimetry was performed in most of the patients because of suspected disproportion, or an intervention had been required in a previous labour. Of those patients that had previous CS and were now exposed to the trial of labour, over 80% delivered spontaneously, and less than 20% required an operative vaginal delivery. This is in agreement with previous studies [6, 13]. Of those women that had had a previous operative vaginal labour, only 5% underwent repeated vaginally assisted delivery. This may have been due to the fact that the patients were chosen for the trial of labour correctly irrespective of the previous operative delivery.

There were no statistically significant differences between the size of the maternal inlet or outlet in the spontaneous and the operative vaginal delivery groups. When patients were divided into subgroups according to the infant weight, the maternal inlet was 4.7% and the outlet was 5.1% larger in the infant weight ≥3700 g subgroup among those who delivered spontaneously compared to those vaginally assisted. The duration of the first stage of the delivery was longer in the smaller infant group, whereas the second stage was shorter than in the larger infant group. In the two delivery subgroups, the duration of the second stage of the delivery was significantly longer in operative vaginal delivery group than in spontaneous vaginal delivery group. The Apgar scores were acceptable in all delivery groups referring to the fact that both spontaneous and operative vaginal deliveries were uncomplicated and severe shoulder dystocia was not present. However, the Apgar 1-minute scores were lower in the operative vaginal delivery group than in spontaneous vaginal delivery group when infant weight was <3700 g. These results refer to the fact that operative vaginal delivery increases the time of the second stage of the delivery and decreases the Apgar 1-minute scores.

The ROC curve analysis for maternal pelvic inlet and outlet revealed that both inlet and outlet had only a fair prognostic value in predicting the mode of the vaginal delivery. The poor predictive value of pelvimetry to predict protracted labor is a well-known fact from previous studies, whereas the evidence on the need of vaginal operative deliveries is less extensively evaluated [3].

The study had some limitations. The data did not reveal in detail the fluency of the operative deliveries. As mentioned earlier, no severe dystocia was present as reflected in the one-minute Apgar scores. Therefore, it was not possible to evaluate the influence of the pelvic dimensions on the severe dystocia. For that kind of study, the cohort examined here study is too small due to the rare incidence of severe dystocia [14]. Accordingly, due to the retrospective nature of the study, no blinding was present, and the caregivers were aware of the pelvimetric measurements during the labour. Furthermore, the study women were at high risk of operative deliveries due to the inclusion criteria, but in clinical care, this would be the group eligible for pelvic assessment before delivery.

In conclusion, our study revealed that maternal bony pelvic dimensions, either pelvic inlet or outlet, were not associated with the need for operative vaginal deliveries. It was more likely that other factors related to the maternal perineal soft tissue, maternal resources, and the passenger were the reasons leading to operative vaginal deliveries. Subsequently, we cannot recommend that caregivers use pelvimetric measurements to predict the outcome of the second stage of the labour. Observational studies with larger cohorts would be needed, if one wished to investigate whether the maternal bony pelvic size has any effect on severe dystocia. In addition, the three-dimensional shape of the bony pelvis and the soft tissues are worth considering in future studies.

## Figures and Tables

**Table 1:** Demographic data of the patients (*N* = 226).

	Spontaneous vaginal delivery	Operative vaginal delivery	*P*-value
Patients	184	42	
Age ± SD (min.–max.) years	28.3 ± 4.7 (19–40)	26.8 ± 4.7 (18–37)	0.75
Parity, nulliparous/multiparous	74/110	33/9	<0.01
Weight ± (min.–max.) kg	68.1 ± 15.5 (43–150)	67.6 ± 16.1 (46–103)	0.87
Height ± (min.–max.) cm	164 ± 5.6 (148–177)	163 ± 5.5 (154–176)	0.92
Body mass index ± (min.–max.)	25.4 ± 5.3 (17–52)	25.4 ± 5.5 (18–39)	0.98
Infant weight ± (min.–max.) g	3750 ± 530 (2210–5120)	3760 ± 380 (2915–4680)	0.80
Labour induction *N* (%)	136 (74%)	30 (71%)	0.78
Labour augmentation *N* (%)	141 (77%)	40 (96%)	0.02

**Table 2:** Descriptive data of the patients subdivided according to the route of delivery and infant weight.

Route of delivery	Inlet mm mean (SD)	Outlet mm mean (SD)	First stage of the delivery, minutes mean (SD)	Second stage of the delivery, minutes mean (SD)	Infant weight g mean (SD)	Apgar 1-minute mean (SD)
All (*n* = 226)	401 (22)	361 (20)	519 (284)	50 (38)	3750 (509)	8.6 (1.1)
Spontaneous vaginal delivery (*n* = 184)	402 (22)	362 (20)	483 (282)	40 (31)	3750 (534)	8.6 (0.95)
Operative vaginal delivery (42)	399 (21)	357 (18)	615 (280)	94 (31)	3760 (378)	8.2 (1.4)
*P*-value	0.23	0.84	0.66	<0.01	0.80	0.06

Infant weight < 3700 g (*n* = 101)	392 (19)	351 (17)	521 (303)	49 (33)	3300 (291)	8.5 (1.0)
Spontaneous vaginal delivery (*n* = 82)	391.5 (18)	352 (17)	515 (309)	40 (29)	3270 (302)	8.7 (0.7)
Operative vaginal delivery (*n* = 19)	393 (19)	347 (15)	543 (282)	85 (27)	3430 (193)	7.8 (1.8)
*P*-value	0.23	0.93	0.56	0.01	0.61	<0.01

Infant weight ≥ 3700 g (*n* = 125)	409 (20)	370 (18)	501 (267)	51 (41)	4120 (318)	8.6 (1.1)
Spontaneous vaginal delivery (*n* = 102)	410 (20)	370 (18)	457.1 (256)	40 (34)	4130 (327)	8.6 (1.1)
Operative vaginal delivery (*n* = 23)	402 (22)	365.5 (16)	675 (270)	102 (32)	4030 (259)	8.6 (0.9)
*P*-value	0.37	0.31	0.65	<0.01	0.78	0.82

SD: standard deviation.
